# Machine learning-enabled regional multi-hazards risk assessment considering social vulnerability

**DOI:** 10.1038/s41598-023-40159-9

**Published:** 2023-08-17

**Authors:** Tianjie Zhang, Donglei Wang, Yang Lu

**Affiliations:** 1 Environmental Research Building, Department of Computer Science, Boise State University, Boise, ID 83725 USA; 2 Environmental Research Building, Department of Civil Engineering, Boise State University, Boise, ID 83725 USA; 3 Boise State University, 1910 University Drive, Boise, ID 83725-2060 USA

**Keywords:** Natural hazards, Environmental social sciences, Climate-change policy

## Abstract

The regional multi-hazards risk assessment poses difficulties due to data access challenges, and the potential interactions between multi-hazards and social vulnerability. For better natural hazards risk perception and preparedness, it is important to study the nature-hazards risk distribution in different areas, specifically a major priority in the areas of high hazards level and social vulnerability. We propose a multi-hazards risk assessment method which considers social vulnerability into the analyzing and utilize machine learning-enabled models to solve this issue. The proposed methodology integrates three aspects as follows: (1) characterization and mapping of multi-hazards (Flooding, Wildfires, and Seismic) using five machine learning methods including Naïve Bayes (NB), K-Nearest Neighbors (KNN), Logistic Regression (LR), Random Forest (RF), and K-Means (KM); (2) evaluation of social vulnerability with a composite index tailored for the case-study area and using machine learning models for classification; (3) risk-based quantification of spatial interaction mechanisms between multi-hazards and social vulnerability. The results indicate that RF model performs best in both hazard-related and social vulnerability datasets. The most cities at multi-hazards risk account for 34.12% of total studied cities (covering 20.80% land). Additionally, high multi-hazards level and socially vulnerable cities account for 15.88% (covering 4.92% land). This study generates a multi-hazards risk map which show a wide variety of spatial patterns and a corresponding understanding of where regional high hazards potential and vulnerable areas are. It emphasizes an urgent need to implement information-based prioritization when natural hazards coming, and effective policy measures for reducing natural-hazards risks in future.

## Introduction

In recent years, natural hazards have become severe threats to society and continued to have a heavy toll on human being. Approximately 45,000 people globally (representing around 0.1% of global deaths) died yearly from natural disasters over the past decade^1^. The United States (US) had sustained 341 weather and climate disasters since 1980, where the total cost of these events exceeded $2.475 trillion, and overall damages/costs reached or exceeded $1 billion (reported by National Centers for Environmental Information, www.ncei.noaa.gov/access/billions). In addition, the previous study showed that over half (57%) of the US structures (office buildings, community houses, schools, hospitals, etc.) were built in hazard hotspots, and about 1.5 million structures were located in hotspots of two or more natural hazards^2^. There was growing awareness of the fact that different hazards could happen simultaneously or successively which would amplify their overall impact on communities^3^. Multi-hazards were defined based on this phenomenon^4^, which could result in a higher number of fatalities, injuries, and displacement of people compared to a single hazard^5^. It could also exacerbate pre-existing vulnerabilities and inequalities, such as poverty, lack of access to resources, or inadequate infrastructure^6^. Moreover, multi-hazards could create complex and interdependent challenges for emergency management officials and policymakers, who must balance competing priorities and coordinate responses across multiple agencies, jurisdictions, and sectors^7^. These challenges highlighted the need for effective multi-hazards risk assessment to help communities better prepare for and respond to natural hazards.

A thorough understanding of hazard risk was essential for devising and implementing effective measures to mitigate and reduce the impacts of hazards^8^. Hazard risk was widely recognized as a combination of three typical components: Hazards, Exposure, and Vulnerability^9^. Hazards refer to potentially destructive physical phenomenon. Exposure refers to the location, attributes, and value of assets that are important to communities and that could be affected by hazards. Vulnerability is defined as people, assets, or a system’s susceptibility to the impacts of hazards, which is often interchangeably used with susceptibility^10^. For Vulnerability, it can be categorized into two dimensions: physical vulnerability and social vulnerability^11^. The term social vulnerability encompasses various dimensions and is specific to a particular context and geographical situation^12^. In the following paragraphs, we will delve into the explanation of this concept and its importance in multi-hazards risk assessment. Additionally, some research defines hazard risk as a combination of hazard potential and vulnerability^13^. In this study, we have combined the assessment of hazard aspects and vulnerability, with a specific emphasis on social vulnerability, to evaluate multi-hazards risk. By considering both hazard potential and social vulnerability, comprehensive hazard risk assessments can identify high-risk areas, prioritize resources, and inform decision-making processes for risk reduction strategies efforts.

The field of multi-hazards research has predominantly emphasized the physical aspects of natural hazards, while paying inadequate attention to the social dimensions of human-hazard interaction. As a result, the assessment of multi-hazards risk has been incomplete and lacking a comprehensive understanding^14^. In the context of multi-hazards, the term social vulnerability refers to the inherent characteristics of social systems that render human societies more or less susceptible to harm and contribute to varying capacities to withstand and recover from impacts^15,16^. It could be influenced by factors like population, the average age in a neighborhood, and the typical housing structure in an area^17^. Social vulnerability offers a comprehensive framework to understand the interactions between populations and the various natural hazards they encounter^18^. It has been evidenced that not all communities are equally equipped to prevent, respond to, and recover from multi-hazards. For example, communities with high proportion of poor population would suffer more from multi-hazards due to lower socio-economic resilience^19^. It is therefore important to take social vulnerability into consideration for a more holistic understanding of regional risks and their differential impacts on various communities when doing multi-hazards risk assessment. As a multidimensional measure of a community's sensitivity to natural hazards and their capacity to anticipate, cope with, resist and recover from the adverse impacts of hazards^20^, social vulnerability enables the identification of hotspots where vulnerable populations that may be disproportionately impacted by hazards and helps to inform the development of targeted strategies to reduce their vulnerability. Current research continues to explore the intersection of multi-hazards and social vulnerability. There have been a lot of attempts to quantify social vulnerability. The most often used calculation method is established by S. L. Cutter and C. Finch^21^, which is a classic, data-driven approach. Recently, this method has then been widely adopted in different types of hazards and countries, such as the Philippines^22^, China^23,24^, Japan^12^, and South Korea^25^.

Multi-hazards risk assessment approaches are still in its infancy period and deserve further development^26^. One key challenge in multi-hazards risk assessment is the need to account for the interactions and interdependencies between different hazards, as well as the vulnerabilities of different systems and communities to these hazards. This requires the use of advanced modeling and simulation techniques, as well as the integration of data from multiple sources and disciplines. Over the last ten years, machine learning has played an increasingly important role in multi-hazards risk assessment to improve the accuracy and speed of identifying and predicting hazardous events. Various machine leaning-based models have been used in risk assessment of multiple hazards. For example, Alessandro Rocchi et al. used the KM clustering algorithm to construct a risk assessment of the combined effects of flooding and earthquakes in Italy. They identified the primary priority of intervention of the study area and delivered helpful information for stakeholders^27^. Additionally, Thimmaiah Gudiyangada Nachappa used support vector machine (SVM) and RF to produce multi-hazards (flooding and landslides) exposure maps for the Federal State of Salzburg, Austria. The results of this study prepared the local planners and managers useful information for the risk areas^28^. Hamid Reza pourghasemi et al. used a RF model to assess flooding, landslides, and forest fire susceptibility in Shiraz city, Iran. Area under ROC curve (AUC) of the model to correctly predict the occurrence or non-occurrence of these three types of hazards reached 0.834, 0.939, and 0.943, respectively^29^. Studies above have tried to apply machine learning models in natural hazards risk assessment which helped to identify risk hotspots at the level of individual countries, sub-national and regional areas.

Despite continued efforts to enhance multi-hazards risk assessments, accurately quantifying social vulnerability and its interplay with multiple hazards remains a complex issue. In addition, the potential of machine learning-enabled approaches to explore the relationship between multi-hazards and social vulnerability has yet to be fully realized. To address this research gap, this study conducts a multi-hazards risk assessment considering social vulnerability using machine learning techniques, with a focus on the state of Idaho in the US as the feasibility study. This work identifies the most widespread hazards in the state, including flooding, wildfires, and seismic, and collects relevant datasets for each type of hazard and social vulnerability. Five machine learning-based models are employed to automatically map three hazards and social vulnerability levels. Finally, the study integrates social vulnerability with multi-hazards to assess their combined impact on different cities. Overall, this study contributes to the ongoing effort to improve emergency management by providing a novel approach to quantifying and understanding the interaction between multi-hazards and social vulnerability using machine learning techniques.

## Methodology

### Overall procedure and study area

Idaho is the 14th largest state and located in the northwestern region of the US. Geographically, this state extends from 44.2405° N to 49.0000° N latitude and from 111.0439° W to 117.2430° W longitude, accounting for a spatial extent of approximately 216,000 km^2^, with a land area of 213,000 km^2^and 3000 km^2^ of water. Administratively, Idaho contains 144 municipalities, and according to the US Census Bureau, the current population of Idaho is estimated to be approximately 1.8 million, most is concentrated in the southwestern part of the state.

Geological (e.g., seismic) and hydro-meteorological hazards (e.g., flooding, wildfires) continuously threaten Idaho’s development and safety. According to the national risk index (US, https://hazards.fema.gov/nri/), Idaho ranked 24th out of the 50 states in overall hazards risk. In addition, some historical and recent natural hazards have generated devastating losses in Idaho. For instance, 2007 Murphy Complex Fire burned more than 650,000 acres in southwestern Idaho, making it one of the largest wildfires in state history. The 2017 Soda Springs Earthquake, a magnitude 5.3 earthquake struck near Soda Springs, Idaho, caused severe damage to buildings and infrastructure. The 2018 flash flood in Horseshoe Bend caused over $1 million in economic losses, and over 100 residents were forced to evacuate their homes.

**Figure 1:**
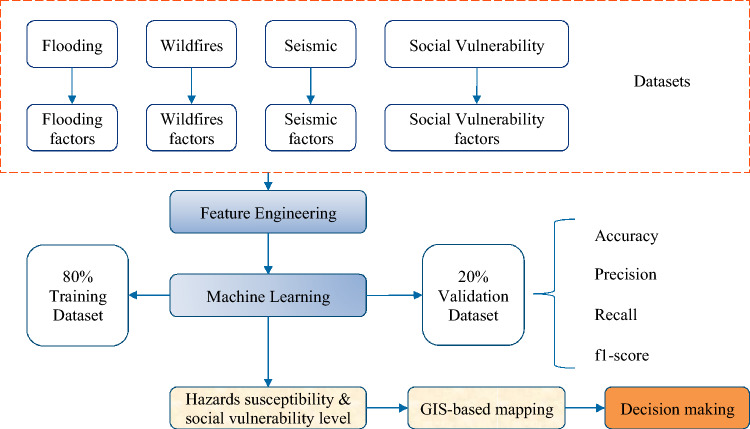
Flowchart of the methodology used for machine learning-based framework for multi-hazards risk assessment considering social vulnerability.

The methodology of this study is presented in Fig. 1. Firstly, the datasets of flooding, wildfires, seismic and social vulnerability covering 84 watersheds and 144 cities in Idaho^30^, are collected from the publicly accessible datasets and government reports. Then, indicator engineering, including invalid data eliminating, scaling, and downsizing, is applied to clean and normalize the data that will improve the performance of machine learning-enabled models. Additionally, the dataset is split into training data (80%) and validation data (20%). The hazards and social vulnerability maps are built based on the processed data. Five machine learning-based models are utilized and compared to predict the damage level caused by each hazard and classify the social vulnerability level automatically. Finally, each area's multi-hazards and social vulnerability level are imported in spatial analyst tool of ArcGIS Pro to build the multi-hazards risk assessment map.

### Dataset

In this study, we are focusing on three types of natural hazards (flooding, wildfires, and seismic activity), as they have accounted for over 86% of all disaster declarations in Idaho since 1954^31^. Due to the high concentration of people, critical infrastructure, and facilities in certain areas, these regions are at greater risk of suffering fatalities and economic losses from multiple natural hazards, particularly in urban areas^32^. Therefore, we are using two open-source datasets that cover information on life and property, as well as population within each watershed, to characterize the potential to these three types of natural hazards and social vulnerability in Idaho. The hazards dataset is obtained from the Idaho Office of Emergency Management (Idaho OEM), while the social vulnerability dataset is obtained from the American Community Survey (ACS) of the US Census Bureau.

Selecting and gathering appropriate variables to serve as inputs is a pivotal stage in any analysis, as it can significantly impact the accuracy and relevance of the resulting outputs. Featuring engineering are utilized based on the downloaded dataset and related instructions. For flooding, we use a series of inputs which include the population at risk of flooding basins, essential facilities in the floodplain, dams of concern, and levees. It should be noted that the levees and hazardous dams are included because the presence of them are considered to be risk factors to flash flooding^33,34^. The inputs used for wildfires include the overall population of the watershed and the number of structures within the wildland-urban interface of each watershed. All these relative risk factors to communities and ecosystems are identified by Idaho department of lands. For seismic, an important input is peak ground acceleration^35^, which is a predicted measurement of ground motion that may be equal to or exceeded 2% annually over a 50-year period. We consider the proportion of high peak ground acceleration in this paper. Also, population and essential facilities in each watershed are selected. For social vulnerability evaluation, indexes based on composite indicators, such as the Human Development Index^36^, the Prevalent Vulnerability Index^37^, or the Social Vulnerability Index (SoVI)^38^, are used to quantify social vulnerability to natural hazards. The SoVI remains the leading conceptual framework among them to assess social vulnerability. Thus, we take SoVI to quantify the social vulnerability of Idaho at county subdivision scale. 27 indicators covering demographic attributes, family structure, education and language, housing and transportation, employment, and economic status are selected.

Overall, Table 1 provides a list of variables selected in each field, the source and abbreviation used in this paper. The choice of these indicators that we considered was based on thorough literature reviews and in-depth discussions with experts in related fields. Also, we prioritize the use of open and accessible data to ensure the analysis can be easily replicated. As hazards are rarely contained within political boundaries, several adjacent communities may be in danger from the same hazard^39^. We assume that the cities located in the same watershed have the same natural hazards potential to be consistent with the social vulnerability assessment at county subdivision scale. This resolution is enough to represent the hazards potential and social vulnerability at a small scale.

**Table 1:** Description of all variables used in this study.

Field	Measure	Source	Abbreviation
Flooding	Population at risk of flooding	Idaho OEM	PF
	Essential facilities in the floodplain		EFF
	Dams of concern		DF
	Levee		LF
Wildfires	Population	Idaho OEM	PW
	Structures in WUI*		SW
	Overall wildfire HUC's level**		HUCW
Seismic	Population	Idaho OEM	PS
	Essential facilities^†^		EFS
	Ground acceleration subtotal percentage of watershed^†^		GAS
Social vulnerability	Median housing value	ACS	MDHSEVAL
	Hospitals per capita		HOSPTPC
	Median gross rent		MDGRENT
	Median age		MEDAGE
	Per capita income		PERCAP
	People per unit (average household size)		PPUNIT
	Percent population under 5 years or 65 and over		QAGEDEP
	Percent Asian		QASIAN
	Percent Black or African American Alone		QBLACK
	Percent unemployment for civilians in labor force 16 years and over		QCVLUN
	Percent less than high school education for population over 25 years and older		QEDLESHI
	Percent speaking English as a second language with limited proficiency		QESL
	Percent employment in construction and extraction industry		QEXTRACT
	Percent children living in married couple families		QFAM
	Percent Female		QFEMALE
	Percent female participation in labor force		QFEMLBR
	Percent female headed households (out of unmarried-partner households)		QFHH
	Percent Native American (American Indian and Alaska Native alone)		QINDIAN
	Percent mobile homes		QMOHO
	Percent housing units with no car		QNOAUTO
	Percent population without health insurance		QNOHLTH
	Percent population living in nursing facilities/skilled nursing facilities		QNRRES
	Percent poverty		QPOVTY
	Percent renters (percent out of total occupied housing units)		QRENTER
	Percent households earning over $200,000 annually		QRICH
	percent employment in service industry		QSERV
	percent Hispanic		QSPANISH
	Percent households receiving social security benefits		QSSBEN
	Percent unoccupied housing units		QUNOCCHU

*Idaho OEM* Idaho office of emergency management, *ACS* American community survey.

*Wildland-urban interface.

**Hydrologic units code.

^†^Within 25 miles of a fault.

### Data processing

**Figure 2:**
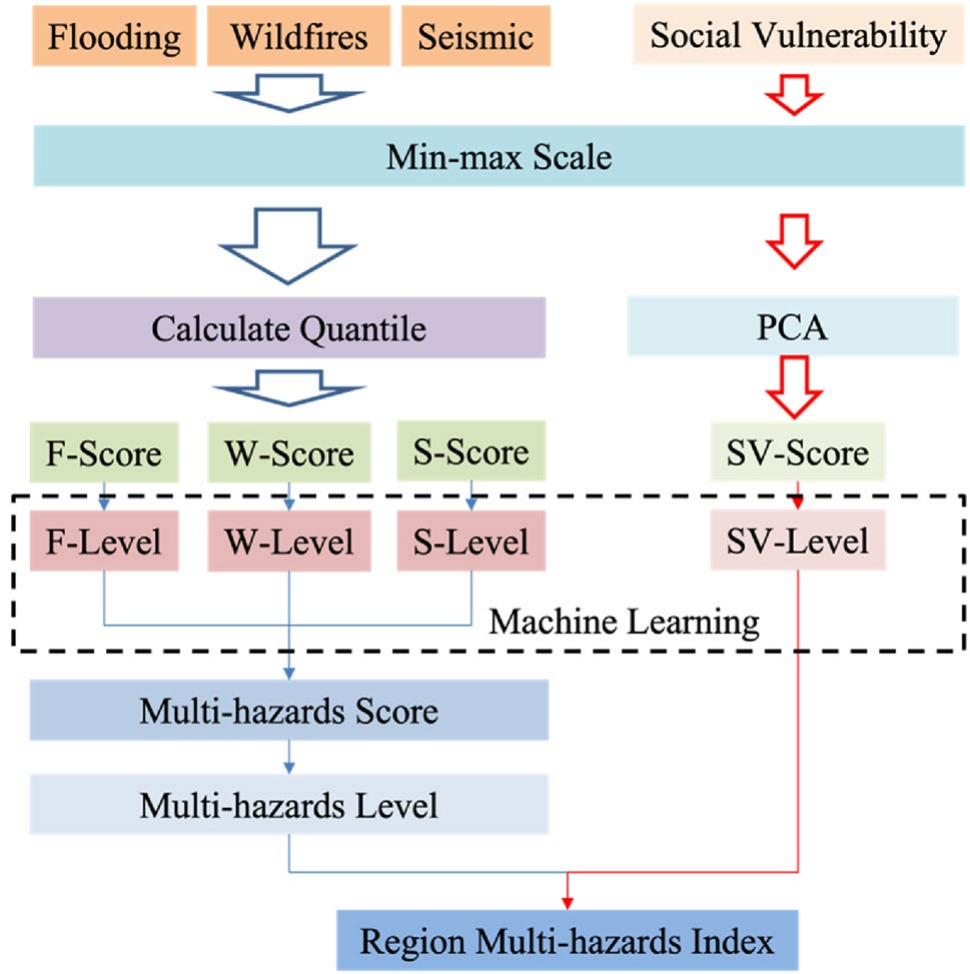
Data preprocessing and annotation process.

The data preprocessing procedure includes removing outlier data, scaling, and PCA. Outliers can be identified as data points that fall outside a specified range. The equation shown below is one common approach to identify the outlier data.

$$Outliers\in \{x<{Q}_{1}-1.5\times IQR\,or\,x>{Q}_{3}-1.5\times IQR\}$$

where ${Q}_{1}$ and ${Q}_{3}$ represent the first and third quartile of the data, respectively, IQR is the interquartile range which is equal to ${Q}_{3}-{Q}_{1}$. After removing the outliers, min–max scaling is applied to the flooding, wildfires, and seismic datasets. PCA is also used to remove the multicollinearity between different factors. Figure 2 illustrates the overall procedure of data processing.

To identify the hazard level of Idaho, the indicators in the hazard datasets (e.g., Population at risk of flooding, Essential facilities in the floodplain, Dams of concern, and levee in flooding) are standardized using the min–max standardization method, as shown in Eq. (1), which generates variables between 0 and 1.

1 $$X=\frac{x-{x}_{min}}{{x}_{max}-{x}_{min}}$$

where X is the scaled data, x is the original data, ${x}_{min}$ is the minimum value in the selected indicator column, ${x}_{max}$ is the largest value in the selected indicator. By doing this, the indicators can be resized to the same scale and be considered equally in the analysis, which is helpful to the classification and evaluation. After standardization, the quantile of each indicator is utilized to calculate the hazard score according to Eq. (2):

2 $$HS=\frac{\sum_{k}^{n}q}{n}$$

where HS stands for the hazard score, n is the number of indicators in the dataset, q is the quantile of the k's indicator for the chosen area, and the quantile is defined and calculated according to Eq. (3):

3 $$P\left[X<x\right]\le q\,\&\,P[X\le x]\ge q$$

where P stands for the possibility, q is the quantile, X is the distribution of the k's indicator, and x is the value when the quantile is q.

This proposed approach is mainly based on the statistical distribution of different factors. Based on the proposed method, each factor can contribute a hazard-related score to the specific hazard. For example, if one region has a larger population at risk of flooding compared to other areas, it would gain a higher HS in flooding because it has a higher quantile compared to others. The HS of each factor in hazard would add together as its hazard score. Then, it would be broken down into three hazard levels: L (low), M (medium), and H (high), as list in Table 2. After getting a region's hazard level for each hazard, the Multi-hazards Score (MHS) is calculated based on Eq. (4).

4 $$MHS=\frac{\sum_{i}^{N}{\Omega }_{i}}{N},{\Omega }_{i}\in \{L=1,M=2,H=3\}$$

where MHS stands for the multi-hazards score, N is the number of hazards, ${\Omega }_{i}$ is the hazard level.

**Table 2:** The hazard level classification.

Hazard	0 < HS < 1/3	1/3 < HS < 2/3	2/3 < HS < 1
Flooding	L	M	H
Wildfires	L	M	H
Seismic	L	M	H

For the social vulnerability, after the data scaling, a PCA is utilized as a data preprocessing method to avoid overfitting problem in machine learning process. Then Analyzing the resulting factors and assess their overall impact on social vulnerability (i.e., whether they increase or decrease social vulnerability) by examining the factor loadings, which indicate the correlation between each individual variable and the entire factor. This analysis should be conducted for each variable in each factor to determine its broad representation and influence. Finally, SoVI is calculated by placing all the components with their directional (positive or negative) adjustments into an additive model. The SV-level (Social Vulnerability Level) is calculated based on the SV-score (SoVI value) which is calculated from results of PCA. The SV-level and Multi-hazards Level are combined to propose a Region Multi-Hazards Index (RMHI) for areas in Idaho. For more details in SoVI calculation, please refer to^40^.

### Machine learning

Natural hazards risk has been widely analyzed and assessed by machine learning-based models like SVM^41^, KNN^42^, LR^43^, RF^44^, and KM^45^. All the models have their unique advantages and drawbacks and there is no indication that a specific model has been utilized in a specific situation^28^. In this study, the choice of machine learning approaches is informed by existing literature reviews and explored established use cases highlighting their application in related research. Additionally, we take careful consideration of the characteristics of our data, such as its volume, dimensionality, and complexity. With all of these considered, five machine learning-based models, including NB, KNN, LR, RF, and KM, are utilized to serve as the prediction models for the hazard level and social vulnerability level assessment. 80% of the data are used in the training process, while the remaining 20% are used in the test step. All the machine learning-based analysis are conducted in Python.

The performance of these machine learning-based models is evaluated by precision (P), recall (R), F_1_ score (F_1_), and accuracy (Acc). These measurements are calculated based on the four parameters, namely true positive (TP), true negative (TN), false positive (FP), and false negative (FN). The calculations of the four measures are shown in the equations below.

5 $$P=\frac{TP}{TP+FP}$$

6 $$R=\frac{TP}{TP+FN}$$

7 $${F}_{1}=\frac{2PR}{P+R}$$

8 $$Acc=\frac{TP+TN}{TP+FP+FN+FP}$$

where P stands for the precision, R is recall, F_1_ is F_1_ score, Acc stands for the accuracy, TP is true positive, TN is true negative, FP is false positive, and FN is false negative.

## Results

**Figure 3:**
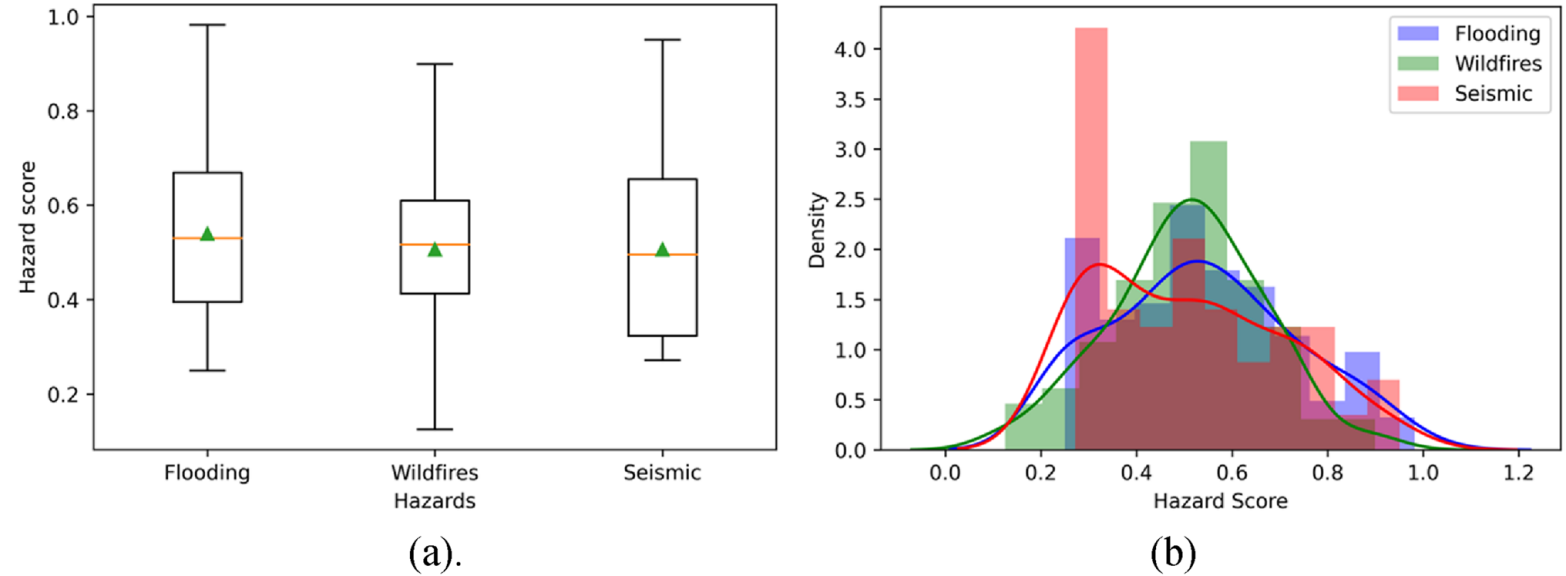
(**a**) The boxplot of the hazard score; (**b**) The histogram of hazard score for three types of natural hazards.

**Figure 4:**
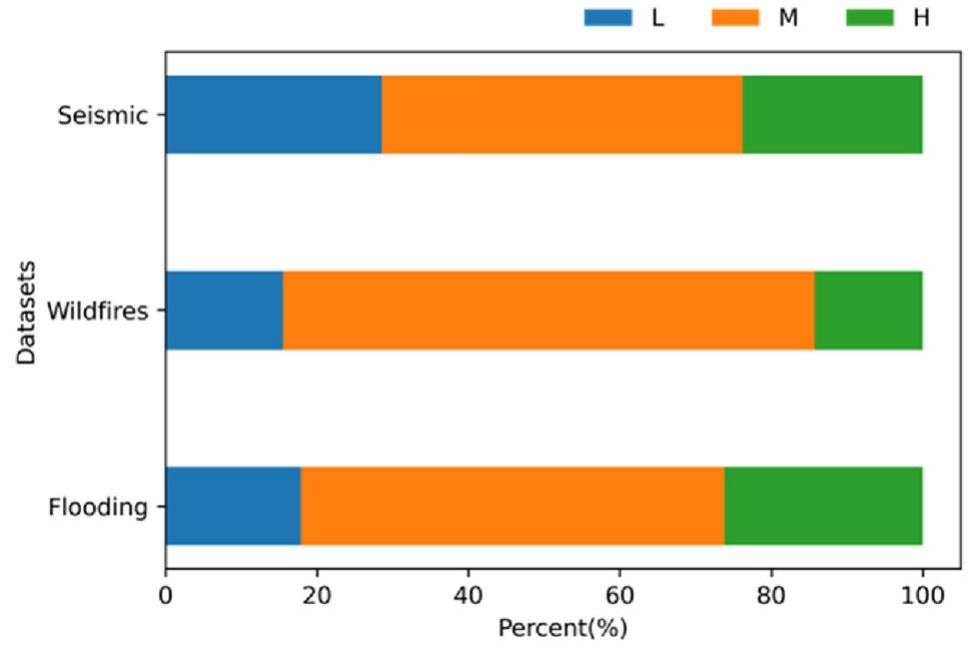
The hazard level distribution of each hazard.

**Figure 5:**
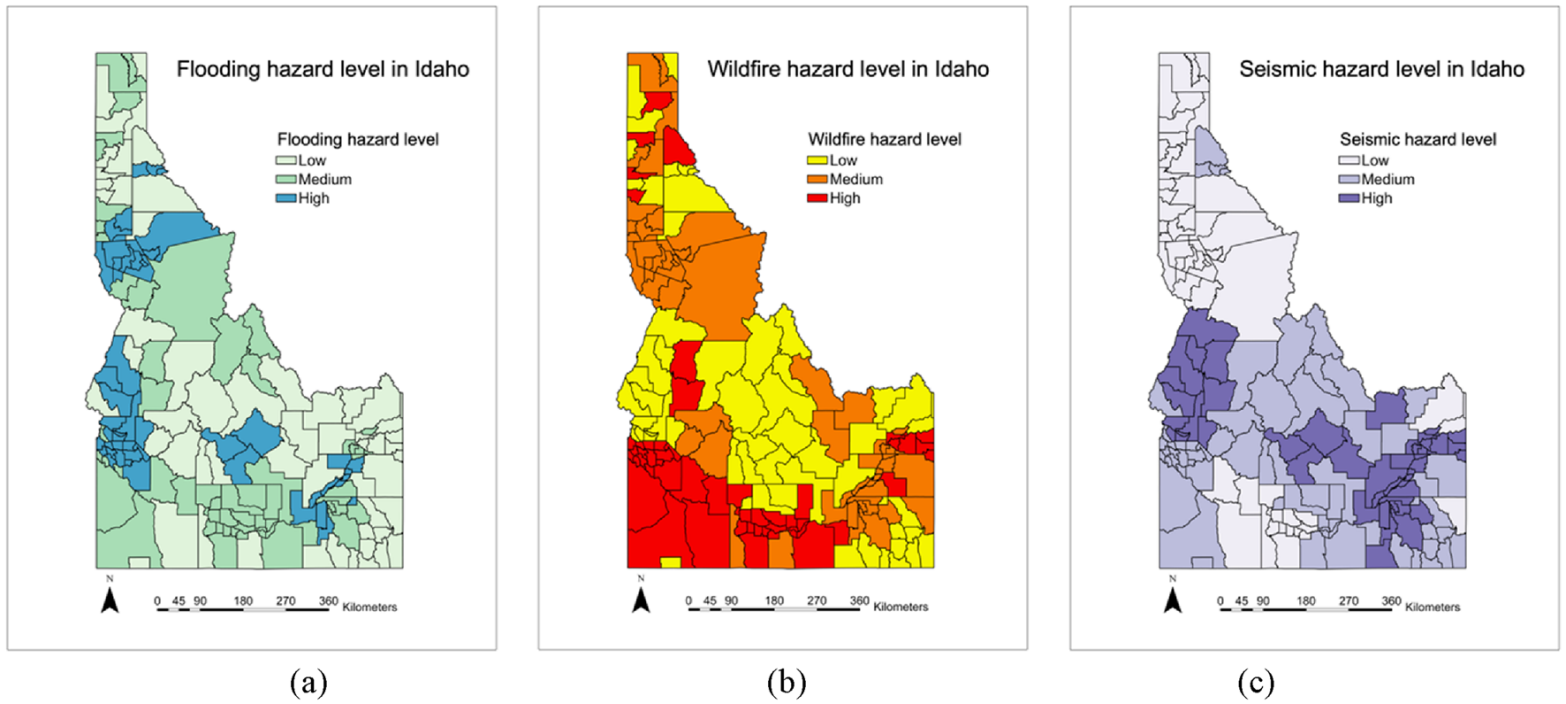
The hazard-level map of three natural hazards (**a**) Flooding; (**b**) wildfires; (**c**) Seismic.

**Figure 6:**
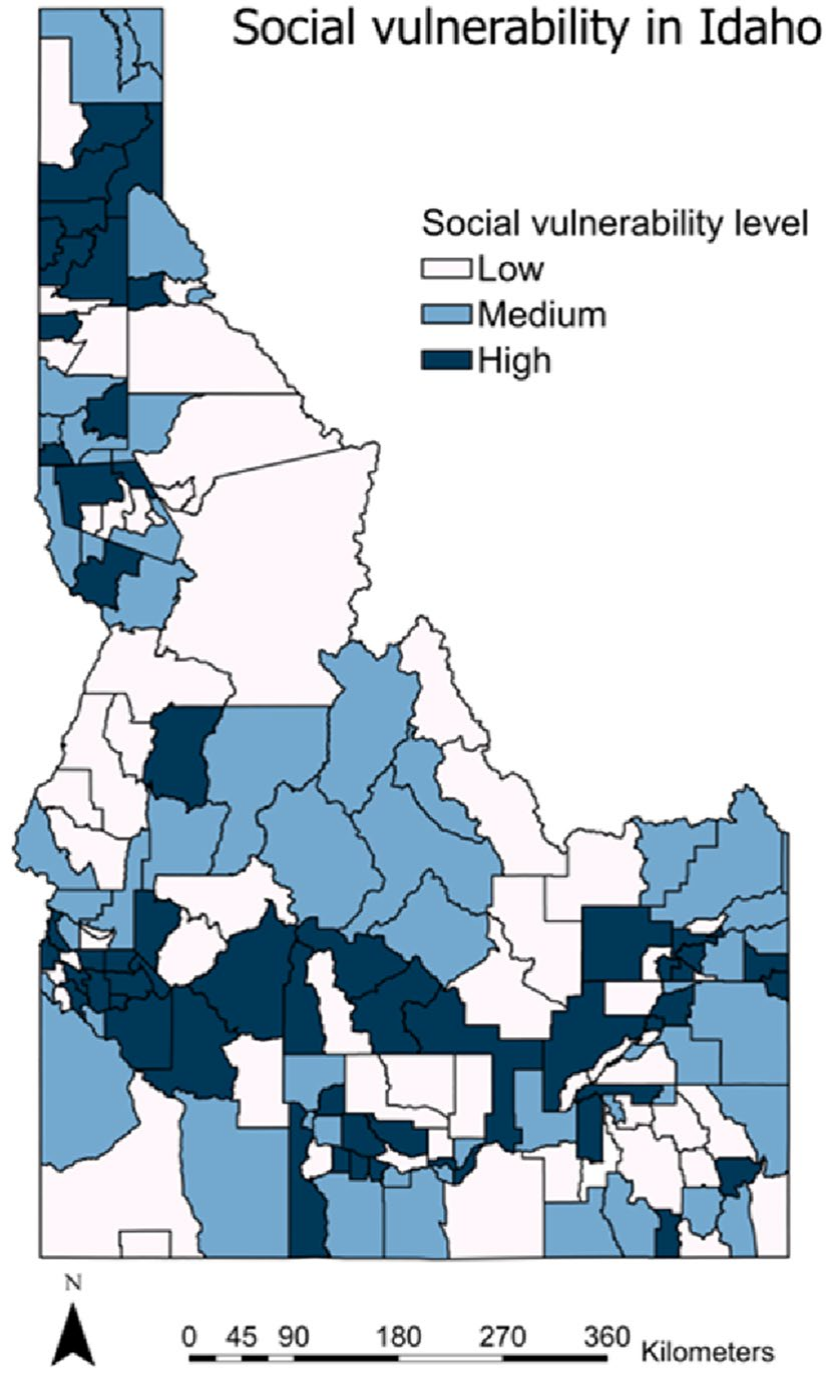
Normalized geospatial social vulnerability scores by county subdivision in Idaho.

**Figure 7:**
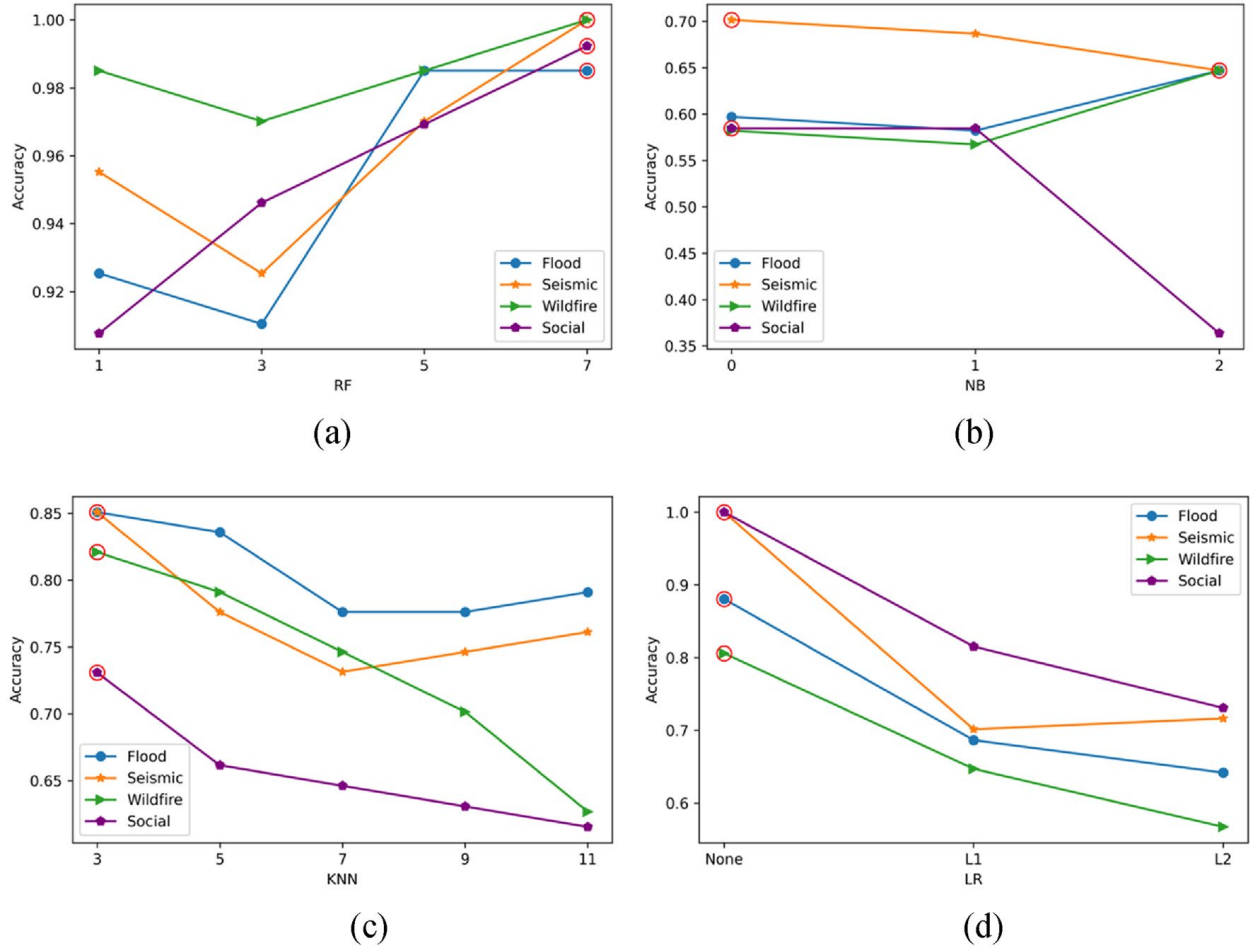
Parameters optimization in different models when utilized in different datasets. (**a**) The number of estimators is the main parameter in RF model; (**b**) The value of alpha is the main parameter in NB model; (**c**) The number of neighbors is the main parameters in KNN; (**d**) The type of penalty is the main parameter in LR model.

**Figure 8:**
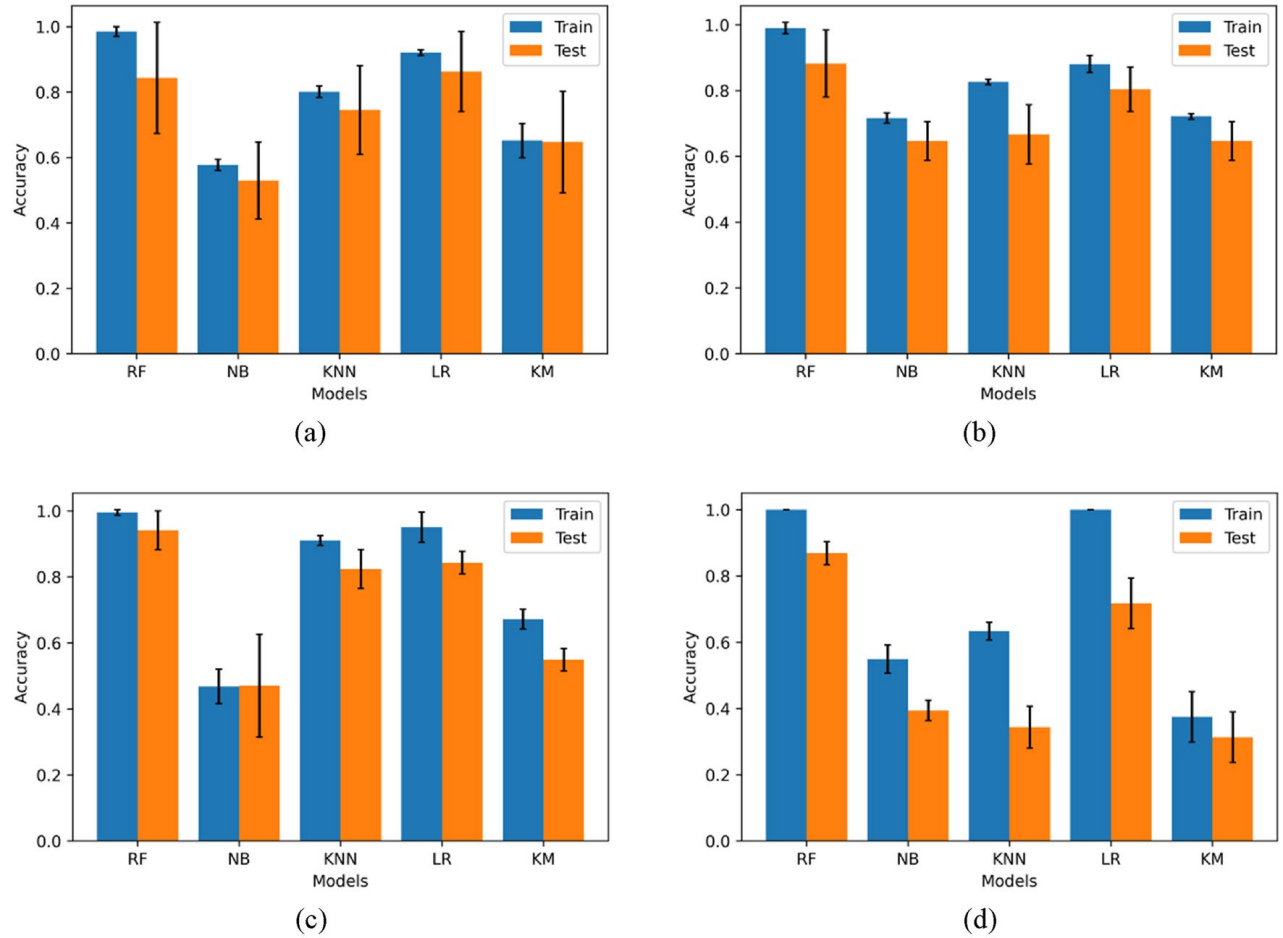
The accuracy score on the train and test data in different datasets: (**a**) Flooding; (**b**) Wildfires; (**c**) Seismic; (**d**) Social vulnerability.

**Figure 9:**
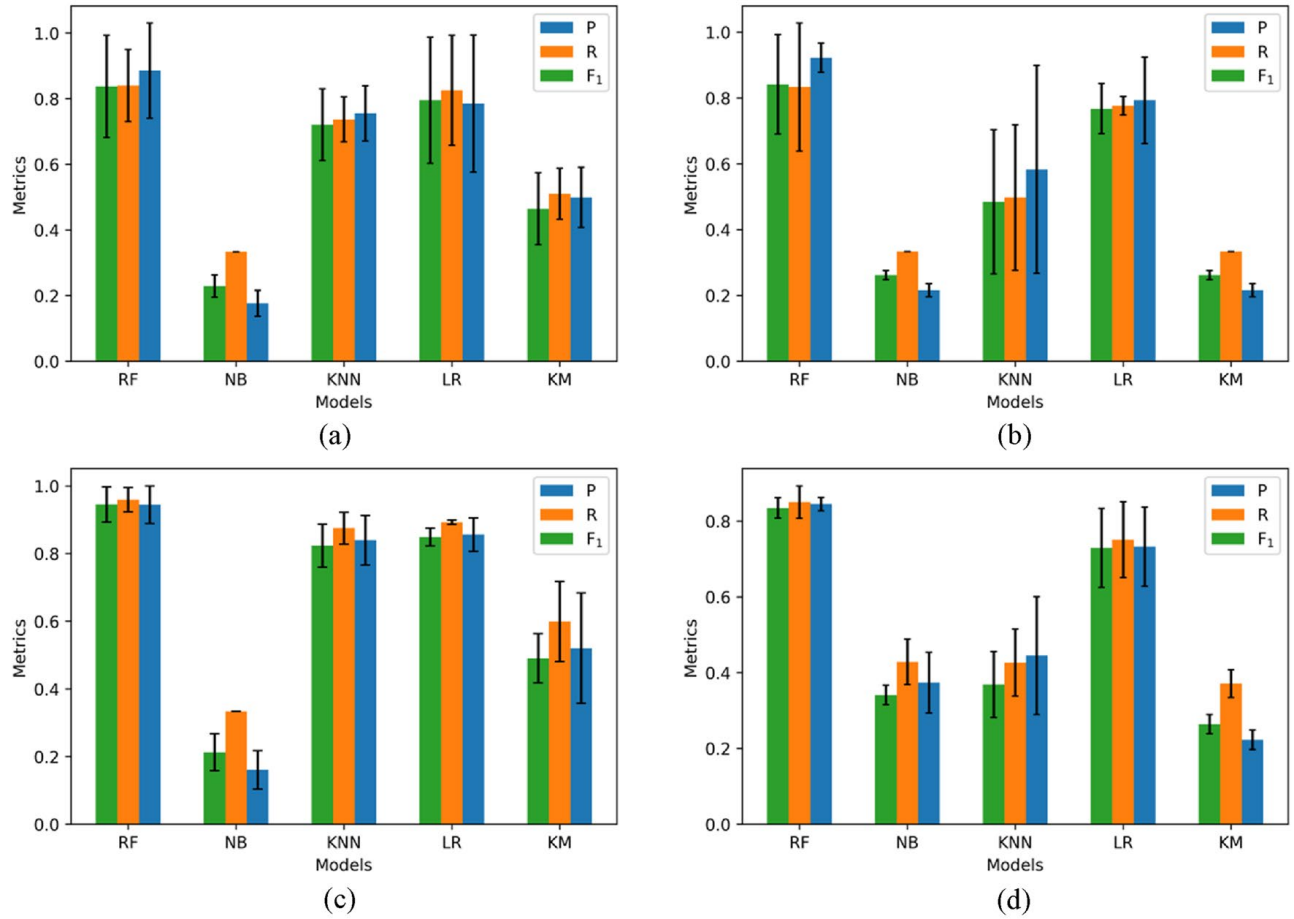
The performance of different machine learning models in different datasets. (**a**) Flooding; (**b**) Wildfires; (**c**) Seismic; (**d**) Social vulnerability.

**Figure 10:**
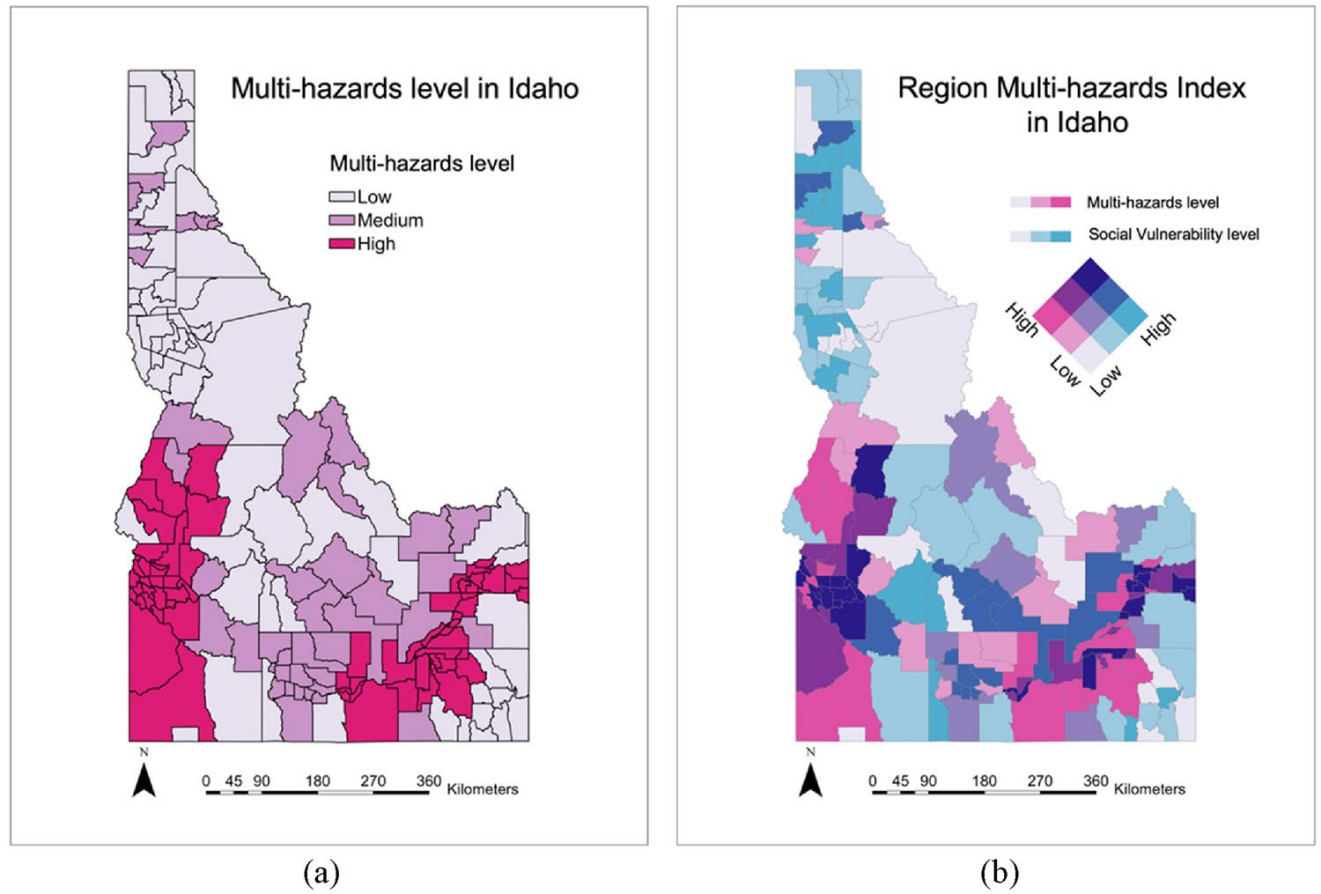
(**a**) Multi-hazards level of different areas in Idaho; (**b**) the RMHI distribution in Idaho.

Figures 3, 4, 5, 6, 7, 8, 9 and 10 show the results of hazards, social vulnerability, spatial distribution of multi-hazards risk, and machine learning measurements. For a visual representation of hazards aspect and social vulnerability, we use different colors to categorize them into 3 classes as low, medium, and high level.

### Hazards level

According to the boxplot shown in Fig. 3a, there is no outliers in the flooding, wildfires, and seismic datasets. It is interesting to find that the average hazard scores of these three hazards are about 0.5, while the average hazard scores of flooding and wildfires are slightly higher than seismic. Figure 3b is a histogram of three hazard scores. It shows that most hazard scores cluster toward the middle of the range, while the rest taper off toward the extreme. The distribution of different levels of each hazard is shown in Fig. 4. It is interesting that the medium level (orange color in Fig. 4) counts the most compared to the low and high levels in all the hazards. The wildfires obtain the largest part of medium level comparing to the others.

The spatial distribution of hazards level is drawn in Fig. 5. The area with high hazard potential level of each hazard is counted. The areas with high level of flooding, wildfires, and seismic account for 16.67%, 13.10%, and 11.90%, respectively. Totally, 19.41% of Idaho cities are found to fall within the potential zone for at least one type of hazard. It is noteworthy that the middle west and southeast areas have high flooding and seismic potential, while southwest areas are with high wildfires level. In other words, some areas are experiencing more than one hazards during the same period. Cities with high hazard level of flooding and seismic, flooding and wildfires, wildfires and seismic, account for 12.94%, 7.06% and 5.29%, respectively. These situations cover 9.08%, 1.87% and 5.29% land of Idaho, respectively. It is worth to note that none of cities is with high hazard level of all three hazards.

### Social vulnerability

After applying the max–min scale, PCA is utilized in the social vulnerability dataset. This is because 27 indicators are included in social vulnerability dataset which may cause overfitting if we don't downsize the number of indicators. PCA can transform the correlated indicators into linearly independent components so that the important information from the indicators is captured. Eight eigen vectors are constructed from the 27 indicators, as shown in Table 3. These eigen vectors are identified as Age, Ethnicity & Education, Wealth, Race & social status, service employment, Nursing service & transportation, and Gender & mobility, based on the dominant variables inside. These eigen vectors count for 70.25% of the total variance of all the data. Then, the impact of each component is adjusted based on their effect on social vulnerability. The positive component direction is associated with increased vulnerability, while the negative component direction is associated with decreasing vulnerability. Normalized and direction-adjusted values of each variable are summed together to determine each city's numerical composite social vulnerability score. Finally, the distribution of normalized geospatial scores is mapped to county subdivisions in Idaho. As shown in Fig. 6, cities with medium or high social vulnerability are mainly located in the southern. In contrast, many areas on the borders (especially the middle parts) have low vulnerability. It is because these areas are covered with forests and have low population density. The most vulnerable cities account for 34.12% of cities in Idaho (covering about 20.80% land in Idaho).

**Table 3:** Idaho county subdivision social vulnerability component summary (the result of PCA).

Component	Cardinality	Name	% Accumulated variance explained	Dominant variables	Component loading
1	+	Age	15.659	MEDAGE	0.842
				QAGEDEP	0.909
				QRENTER	− 0.653
				QUNOCCHU	0.762
				QCVLUN	0.672
				QSSBEN	0.836
2	+	Ethnicity & education	26.356	QHISP	0.726
				QSEL	0.832
				QED12LES	0.813
				QEXTRCT	0.633
3	−	Wealth	36.340	PERCAP	0.866
				QRICH200K	0.677
				MDGRENT	0.682
				MHSEVAL	0.633
4	+	Race & social status	45.215	QNATAM	0.734
				QFAM	− 0.636
				QFHH	0.690
				QPOVTY	0.678
5	+	Service employment	52.911	QPUNIT	0.639
				QFEMLBR	0.819
				QSERV	0.732
6	+	Nursing service & transportation	59.500	QNRRES	0.706
				QNOAUTO	0.715
7	+	Race	65.417	QASIAN	0.691
				QBLACK	0.706
8	+	Gender & mobility	70.251	QFEMALE	0.711
				QMOHO	− 0.667

### Evaluation of machine learning models

Machine learning methods are used and compared to classify the potential of different hazards. By doing this, we can automatically and artificially identify each hazard's potential. Firstly, the parameter in each model is compared to see which one is best suit for different dataset. As we can see from Fig. 7, number of estimators of 7 obtain the best performance among all the datasets. An alpha of 0 works best in Seismic and Social vulnerability data while an alpha of 2 can get the highest accuracy in the Flooding and Wildfires dataset. For the KNN algorithm, the best number of neighbors is 3 for all the datasets while for the LR approach, with the penalty of ‘None’ can help obtain the highest accuracy. KM is a clustering method, and its main parameter is the number of clusters which is 3 in this work. Thus, there is no parameter optimization conducted in the KM algorithm.

To evaluate and compare the performance between different machine learning-based models statistically, each test is run three times. The accuracy of each model on the train dataset and test dataset are calculated and compared, as shown in Fig. 8. The accuracy difference between the train and test datasets can be an index of overfitting. As shown in Fig. 8d, the RF and LR both perform well (the accuracy is 1.0) on the train dataset. However, the accuracy difference between the train and test in the LR is more significant than in the RF. It means the LR in this context has a more severe overfitting problem than the RF. It is noteworthy that the KM algorithm performs poorly in the social vulnerability dataset compared to other datasets. It is mainly because there are more indicators in the social vulnerability dataset than in other datasets. In other words, the increasing number of indicators can decrease the accuracy of the KM method as the complexity of clustering is dramatically increased.

To comprehensively describe the models' performance, the precision, recall and F_1_ score are calculated and compared among each model, as shown in Fig. 9. The average precision, recall and F_1_ score of random forest model are 0.842, 0.836, 0.840 for flooding, 0.867, 0.868, 0.854 for wildfires, 0.928, 0.927, 0.908 for seismic, and 0,797,0,789, 0.820 for social vulnerability, respectively. It is noted that the RF algorithm gets the highest precision, recall and F_1_ score among all the models (all these measures are over 0.8), which means it outperforms other classification approaches in all datasets. The KM algorithm is one of the two relatively low-performance models. This is because KM is an unsupervised-learning model. The performance of KM heavily depends on the indicators and whether the indicator can well reflect the level. The other low-performance model in this study is NB. All the three measures values of NB are lower than 0.5.

### Spatial interaction of multi-hazards and social vulnerability

In this work, a RMHI is proposed to show the impact the hazards could have on a particular area. The effects of multiple hazards and the impact of social vulnerability in each area are counted in the RMHI. In other words, this RMHI shows the possible damage that hazards can cause to a specific area based on the social vulnerability of this area. To calculate and map the RMHI, the multi-hazards score is calculated first based on the method in Eq. (4). Then, the multi-hazards potential is leveled based on the score. The spatial distribution of the multi-hazards level is shown in Fig. 10a.

The intersection of multi-hazards and social vulnerability is finally divided into nine zones, the low hazard with low social vulnerability zone, low hazard with medium social vulnerability zone, low hazard with high social vulnerability zone, medium hazard with low social vulnerability zone, medium hazard with medium social vulnerability zone, medium hazard with high social vulnerability zone, high hazard with low social vulnerability zone, high hazard with medium social vulnerability zone, and high hazard with high social vulnerability zone. Among these zones in Fig. 10b, we need to take an insight into those with high hazard level (about 39% of all studied cities) and high social vulnerability (about 33% of total studied cities). From the final multi-hazards risk map, we can see that these areas with high multi-hazards level and social vulnerability are mainly distributed in the southeast and southwest areas, accounting for 15.29% of the study cities. The prioritization mitigation should be considered in these areas. Additionally, these results and findings allow us to forecast the spatial behavior of such multi-hazards events. It can help policymakers, and emergency managers better understand how we should characterize patterns of multi-hazards vulnerability at aggregate scales for comparative use. The results also can support a variety of professionals in better tailoring their mitigation strategies or planning efforts to populations who are most likely to help develop, benefit from, or carry out any actions being implemented. Finally, the results of this research can contribute to ongoing dialogues about potential social inequality of human populations exposed to hazards and what some argue is state or federal subsidization of private property development in high-risk areas through fire suppression spending.

## Conclusions

This work has explained spatial relationships between multi-hazards and social vulnerability in Idaho, US, where flooding, wildfires, and seismic are the most significant natural hazards. Two public open access datasets are used to characterize the distribution situation of flooding, wildfires, seismic and social vulnerability in this study area. The composite SoVI is used to quantify social vulnerability. Machine learning-based models are implemented to predict the natural hazards potential and social vulnerability level, respectively. The combination of multi-hazards level and SoVI deliver quality evidence that increases public awareness, support information-based policymaking in disaster risk management, and prioritize mitigation and resiliency actions that reduce risk to life and property in high-risk areas.

Results show that the RF overperforms other machine learning-based models on precision, recall and F_1_ score, which offers a good promise in both the natural hazards and social vulnerability level classification. The multi-hazards map at the county subdivision level, reveals that most land is not prone to high-level multi-hazards. Cities with high hazards level and cities with high social vulnerability account for about 39% and 33% of total studied cities, respectively. Cities with high multi-hazards risk account for 15.29% of total studied cities. The multi-hazards risk index map can be used for integrated and comprehensive watershed management and land use planning and, consequently, for sustainable development in the study region. In addition to the insights of multi-hazards risk, recognizing the vulnerable or susceptible areas and identifying the main drivers of high social vulnerability level can provide the government and decision-makers more robust information, and assist them in disaster risk reduction.

Although this paper studies the spatial relationship and interactions of multi-hazards and social vulnerability in case study area, similar framework can be performed in other geographical areas to consummate multi-hazards risk assessment from a comprehensive perspective. As this framework can be easily replicated using only public open data, we hope this research will inspire the development of similar models and decision-making tools to identify the highest hazard risk but high social vulnerability areas. Moreover, while the data need to be updated, machine learning-based models can help to update and improve the speed of analysis. For instance, the census information using for estimations of social vulnerability is often updated every five years, machine learning models can help to capture this online updated census information and adjust social vulnerability to natural hazards and even to produce future projections. Also, the information improvement in government reports (e.g., newest edition) can be adjusted and handled quickly via a set of machine learning pipelines. We believe such tools are needed and valuable, it can provide decision-makers with more precise and timely information about potential hazards, which can help them make more informed decisions and take appropriate actions to mitigate the risks.

## Data Availability

The datasets used and/or analyzed during the current study are available from the corresponding author on reasonable request.
